# Planning and performance in teams: A Bayesian meta-analytic structural equation modeling approach

**DOI:** 10.1371/journal.pone.0279933

**Published:** 2023-01-13

**Authors:** Udo Konradt, Alexander Nath, Martina Oldeweme

**Affiliations:** Institute of Psychology, Kiel University, Kiel, Germany; Universiti Malaysia Sabah, MALAYSIA

## Abstract

We meta-analyzed the relationship between team planning and performance moderated by task, team, context, and methodological factors. For testing our hypothesized model, we used a meta-analytic structural equation modeling approach. Based on *K* = 33 independent samples (*N* = 1,885 teams), a mixed-effects model indicated a non‐zero moderate positive effect size (ρ = .31, 95% CI [.20, .42]). Methodological quality, generally rated as adequate, was unrelated to effect size. Sensitivity analyses suggest that effect sizes were robust to exclusion of any individual study and publication bias. The statistical power of the studies was generally low and significantly moderated the relationship, with a large positive relationship for studies with high-powered (*k* = 42, ρ = .40, 95% CI [.27, .54]) and a smaller, significant relationship for low-powered studies (*k* = 54, ρ = .16, 95% CI [.01, .30]). The effect size was robust and generally not qualified by a large number of moderators, but was more pronounced for less interdependent tasks, less specialized team members, and assessment of quality rather than quantity of planning. Latent class analysis revealed no qualitatively different subgroups within populations. We recommend large‐scale collaboration to overcome several methodological weaknesses of the current literature, which is severely underpowered, potentially biased by self-reporting data, and lacks long-term follow-ups.

## Introduction

**Fig 1:**
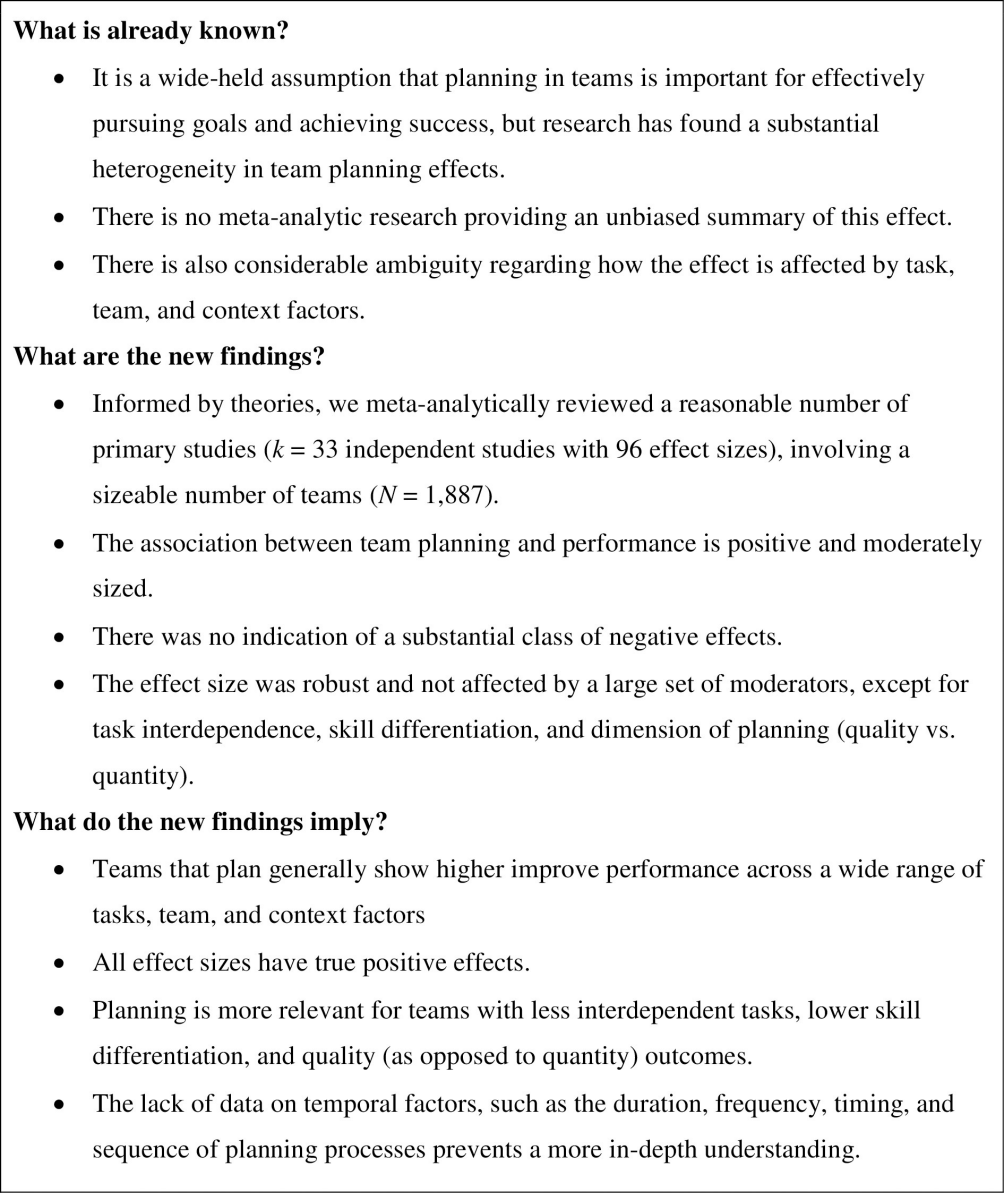
Summary box.

Planning is considered to be an indispensable and essential prerequisite for individuals, teams, and organizations to take purposeful action and to achieve success [1]. In organizational psychology and organizational behavior, team planning generally refers to a group-level process, which is essential for accomplishing tasks, coordinating effort, and for general effectiveness [2]. Among the various benefits of planning are that it can help teams to establish and synchronize goals [3], improve their use of resources, schedule work tasks, and set appropriate deadlines [4]. In addition, planning serves to decide how to measure success and what milestones to set, which have an important function in monitoring and feedback according to several psychological theories of learning, motivation, and leadership. Consequently, theories of team planning (e.g., [5, 6]) and qualitative reviews (e.g., [1, 7]) in the organizational psychology literature suggest that team planning has positive effects on team performance. However, despite its theoretical and practical relevance for team processes and outcomes, we cannot draw robust and broad conclusions. Thus, a meta-analysis is warranted that provides an unbiased summary of the effect of team planning on team performance (Fig 1).

Existing theory and research on team processes suggest that the strength of planning’s effect on performance is conditional on several moderators that affect how teams act, including task, team, and context factors (e.g., [8]; for a review, see [9]). Understanding these conditions is essential to refine existing planning theory and is also important to inform practice and policy in organizations. Thus, the second aim of our research is to provide insights into the moderating role of these boundary factors.

With our study, we also aim to expand the focus on the positive effects of team planning, currently dominant in theory and research, to provide a more differentiated view that allows for the possibility that planning can have negative effects or a null effect on performance in teams. In the context of entrepreneurship research, the effects of planning at the organizational level were found to be heterogeneous, indicating no uniform direction [10, 11]. Studies in other contexts also provide further evidence that there can indeed be negative relationships between team planning and team performance (e.g., [12, 13]). Consequently, the third aim of this study is to advance understanding of team planning by identifying classes of positive and non-positive effects and specifying the conditions under which they occur.

In the present study, we systematically review the literature to explore how planning affects performance in teams and to examine whether there are systematic differences in this relationship. We combined a reasonable number of primary studies (96 effect sizes), involving a sizeable number of teams (*N* = 1,885). We used Bayesian meta-analytic structural equation modeling (MASEM) to improve the statistical power and estimate a true effect more precisely. Drawing on team effectiveness frameworks, we also contribute to the existing literature by analyzing a broad range of moderators that might attenuate or reverse the relationship between planning and performance. Specifically, by aggregating across small studies, which are typical of research in this area, we achieve adequate statistical power to examine factors that moderate the effect of team planning that have never before been addressed in any primary study. We also consider several methodological factors of studies to assess the reliability of the results.

## Theory and hypotheses

### Planning in teams

A review of the organizational literature revealed that team planning has been defined in various ways [14]. In the broadest sense, it refers to team processes—mechanisms by which team inputs are transformed into team outcomes [6]—directed at generating “a proposed sequence of actions intended to accomplish a set goal” ([15], p. 488). Marks and colleagues [16], who divide team processes into transitions, action, and interpersonal processes, regard planning as a transition process, referring to an activity that guides a team’s accomplishment of a goal. To be more specific, team planning involves the decision-making processes of specifying goals and determining the course of action, setting out rules and procedures for handling critical situations, allocating resources and assigning roles, and monitoring progress toward the goal [6, 17, 18]. Moreover, planning is described as “the active and conscious mental simulation of future action sequences intended to direct behavior and optimize the attainment of certain outcomes” [1, p. 214], In this meta-analysis, we define team planning as an interactive transition process taking place among team members, which is aimed at generating, evaluating, and selecting future activities to achieve the team’s goal or objective.

Team planning has been conceptualized in several ways that differ in terms of their focus on timing, function, or content (e.g., [5]). A prevalent conceptualization is that of Weingart [18], who conceptualizes team planning in terms of timing by distinguishing between pre-planning, which takes place before work on the task begins, and in-process planning, which takes place while it is underway. Similarly, Marks and colleagues [16] distinguish between reactive adjustment, where plans are adjusted while working on the task, deliberate planning, in which all the actions needed to complete the task are determined in advance, and contingency planning in which alternative plans are developed when the anticipated conditions change. Despite different foci, all definitions address the core elements of generating, evaluating, and selecting future activities relevant to the team’s goal or objective.

### Planning in teams and performance

Transition processes such as team planning are considered to be a fundamental element in accomplishing tasks and team functioning, and essential for team effectiveness [1, 7, 16, 19]. In organizational psychology and organizational behavior, team effectiveness and team performance are broadly conceptualized in terms of tangible outputs from the team and reactions by team members [20]. Team performance refers to the extent to which teams fulfill the requirements of their job effectively, as identified formally in their job description [21], in terms of quality, functionality, and reliability [22]. Meta-analytic evidence suggests moderate positive effect sizes between transition processes and team performance [23]. Similarly, reflexivity theory [19] and team self-regulation theory (cf. [24]) propose that team planning increases team performance, as it leads to more effective problem solving and enhances coordination. Mumford and colleagues [1] also argue that planning promotes learning and enhances motivation in teams, both of which improve performance. George and colleagues’ research [25] provided empirical evidence for this contention at the organizational level. We therefore offer the following hypothesis:

**Hypothesis 1:** Planning in teams is positively related to performance.

### Moderators

Theories and frameworks of teamwork suggest that a range of factors either directly affect or moderate the relationship between transition processes in teams and performance. However, it remains unclear whether these factors play a significant role in the relationship between team planning and performance. In integrative and ‘meta’ frameworks of team effectiveness (e.g., [8, 26, 27]) moderators are divided into three categories: task, team, and context factors. For each of these categories, research on team planning has suggested that several variables may be relevant. Within frameworks focusing on team effectiveness, factors specific to the task include the type of task, task interdependence, time constraints, and task complexity, because all of these factors are assumed to be important determinants of the relationship between team processes and performance [19, 28–30].

Team factors that influence the planning process may include authority, team size, team familiarity, skill level, and skill differentiation [23, 31–34]. Context factors that can affect the planning process may include knowledge distribution, separate planning sessions, familiarity with the task, the type of planning (i.e., pre-action vs. in-action planning), and guided planning [18, 35–38]. The relevant moderators are summarized in Table 1, together with definitions and corresponding literature.

**Table 1:** Definitions, sources, and coding of moderators.

Category and Moderator		Definition	Source	Coding^a^
*Task factors*				
1	Type of task	Main categories of task, corresponding to McGrath’s [28] task circumplex model	McGrath (1984) [28]	1 = generate, 2 = execute, (3 = choose, 4 = negotiate)
2	Task interdependence	Degree to which the task requires team members to work closely with each other and share material and expertise	Guzzo & Shea (1992) [29]	1 = low, 2 = medium, 3 = high
3	Time constraints	Limitations imposed on the team in terms of the maximum time allowed (deadline) to complete the task	March, 1959, as reported in Shure et al., 1962 [30]	1 = no, 2 = yes
4	Task complexity	Degree of information-processing difficulty in terms of the amount of information and number of operations required for the task, and the interrelations and dynamics involved	Weingart, 1992 [18]	1 = low, 2 = medium, 3 = high
*Team factors*				
5	Authority	Degree of the team’s legitimate power to act/operate in a self-determined way	Langfred, 2005 [31]	1 = semi-autonomous, 2 = self-regulated
6	Team size	Number of team members working on the task	LePine et al., 2008 [23]	Count number
7	Team familiarity	Degree of prior interaction and shared experiences between group members	Harrison et al., 2003 [32]	1 = low, 2 = medium, 3 = high
8	Skill level	Level of team members’ knowledge and ability	Mathieu et al., 2000 [33]	1 = low, 2 = medium, 3 = high
9	Skill differentiation	Level of team members’ specialized knowledge or functional capacity	Lee et al., 2015 [34]	1 = low, 2 = medium, (3 = high)
*Context factors*				
10	Knowledge distribution	Degree to which knowledge or functional capacity is distributed between team members	Stasser & Titus, 1985 [35]	1 = unequal, 2 = equal
11	Planning session	Task for which there is a mandatory planning lesson.	Chen et al., 2005 [36]	1 = no, 2 = yes
12	Task familiarity	Degree to which team members have had experience of the task	Earley et al., 1990 [37]	1 = low, 2 = medium, 3 = high
13	Type of planning	Point at which planning takes place relative to action processes	Weingart, 1992 [18]	1 = pre-action, 2 = in-action
14	Guided planning	Procedure used to guide the planning process.	Smith-Jentsch et al., 2008 [38]	1 = no, 2 = yes
*Methodological factors*				
15	Type of study	Type of design used for the study.	Mitchell, 2012 [39]	1 = correlational, 2 = experimental
16	Dimension of planning	Quantitative or qualitative assessment of team planning	Smith et al., 1990 [41]	1 = qualitative, 2 = quantitative
17	Study setting	Nature of the study setting	Tannenbaum & Cerasoli, 2013 [40]	1 = hypothetical, 2 = real-life
18	Type of planning data	Mean by which planning data were obtained.	Andersson et al., 2017 [42]	1 = self-report, 2 = observation
19	Type of performance data	Means by which performance data were obtained.	Andersson et al., 2017 [42]	1 = self-rated, 2 = other-rated, 3 = task score

Note

^a^ Categories in brackets did not occur

Finally, we also consider whether effect sizes are contingent on methodological moderators such as the type of study, dimension of planning, study setting, and the measures used for both planning and performance (see Table 1). Previous research on teams has indicated that effect sizes are strongly dependent on whether the study is correlational or experimental [39] and conducted in real-life or hypothetical settings [40]. We also examine whether the scales used to measure planning and performance influence the effect sizes in terms of what dimension of planning is used [41] and also what measure [42]. In addition, we examine the methodological quality of each study, using as our criteria the sampling and representativeness (four items), the statistical analyses (two items), reliability and validity of the measures (two items), and the design and fidelity (two items, see S1 Appendix). Thus, we address the following research question:

**Research Question 1:** Does the association between team planning and team performance vary according to the (a) task factors (type of task, task interdependence, time constraints, and task complexity), (b) team factors (authority, team size, development stage, skill level, and skill differentiation), (c) context factors (knowledge distribution, planning session, task familiarity, and treatment), and (d) methodological factors (type of study, dimension of planning, study setting, measure of planning, and measure of performance)?

### Unobserved heterogeneity in the effects of planning on performance

Although team planning has been suggested to have a positive effect on task completion, team processes, emergent states, and performance [3, 7, 19], researchers have found there to be substantial heterogeneity in team planning effects and have also shown planning to have some detrimental effects (e.g., [12, 13]). This suggests unobserved heterogeneity in the sample, where different patterns of effect sizes may belong to different subpopulations and in classes not captured by observable characteristics [43]. Describing these classes and examining how they come about could advance theory and inform practice.

One approach to uncovering these different subpopulations is to consider the risks and potential flaws associated with team planning. Montoya and colleagues [44] outlined five major problems which teams encounter when planning, including a tendency to discuss shared rather than unique information, a bias towards pre-discussion preferences, unevenly distributed participation and contribution to the discussion, excessive cohesiveness, which leads to groupthink, and avoidance of team planning. Those tendencies are regarded as natural team behaviors, which limit their planning capabilities, deplete their resources, and thus make them less effective. This is consistent with resource allocation theory (e.g., [45]), which posits that planning requires time and cognitive resources and might be a distraction, preventing a team from focusing on actions, processes, and task performance [14, 46]. Likewise, Mumford and colleagues [1] argued that team planning can be a demanding and intensive activity; people must therefore be able and willing to invest their limited resources in it. Also, a detrimental effect might be particularly large if the task has a high routine component and follows an algorithm or well-known rules. In fact, the time-consuming and resource-intensive nature of planning is often seen to be at odds with enhancing performance; planning is regarded as a distraction from goal-directed actions and as a reason why resources become depleted. Lei and colleagues [12] provided empirical evidence of this; they showed that planning activities initially increased team performance, but too much planning had adverse effects on task work and subsequent performance. This implies that different patterns of relationships might be expected. When reviewing team planning research in entrepreneurship settings, Gielnik et al. [11] concluded that there were marked differences in effect sizes found in primary studies, suggesting that effect sizes do not follow a specific pattern. In light of this, we expect our sample to show two distinct classes of effect sizes: one with positive effect sizes and another with non-positive effect sizes. Therefore, we hypothesize that:

**Hypothesis 2:** In terms of the relationship between planning and team performance, there are two classes of studies that differ in their mean effect size: One group shows a positive effect size and the other group shows a non-positive effect size.

## Method

### Literature search

The data were initially collected in October through December of 2017 and were augmented by a second collection period in March/April 2021. The data were collected in four ways including published as well as unpublished studies and datasets to minimize publication bias [47]. First, we conducted a systematic keyword search in six electronic databases (PsycInfo, Scopus/ScienceDirect, EBSCO, APA, PubPsych, ERIC). To account for unpublished data and grey literature we additionally searched the databases Google Scholar and SSRN. In sum, this search covered articles, dissertations, books, reports, theses, unpublished datasets, and preprints. Titles, abstracts, and keywords were searched for the following terms: team planning OR planning in teams OR group planning OR planning in groups. We considered studies published during the last 50 years. Second, we checked the studies included in meta-analyses of team processes as well as those included in references in primary studies [2, 23]. Third, we searched for unpublished data via mailing lists and contacted researchers likely to have worked in this area. This search yielded a total of 1,888 matches.

### Inclusion and exclusion criteria

**Fig 2:**
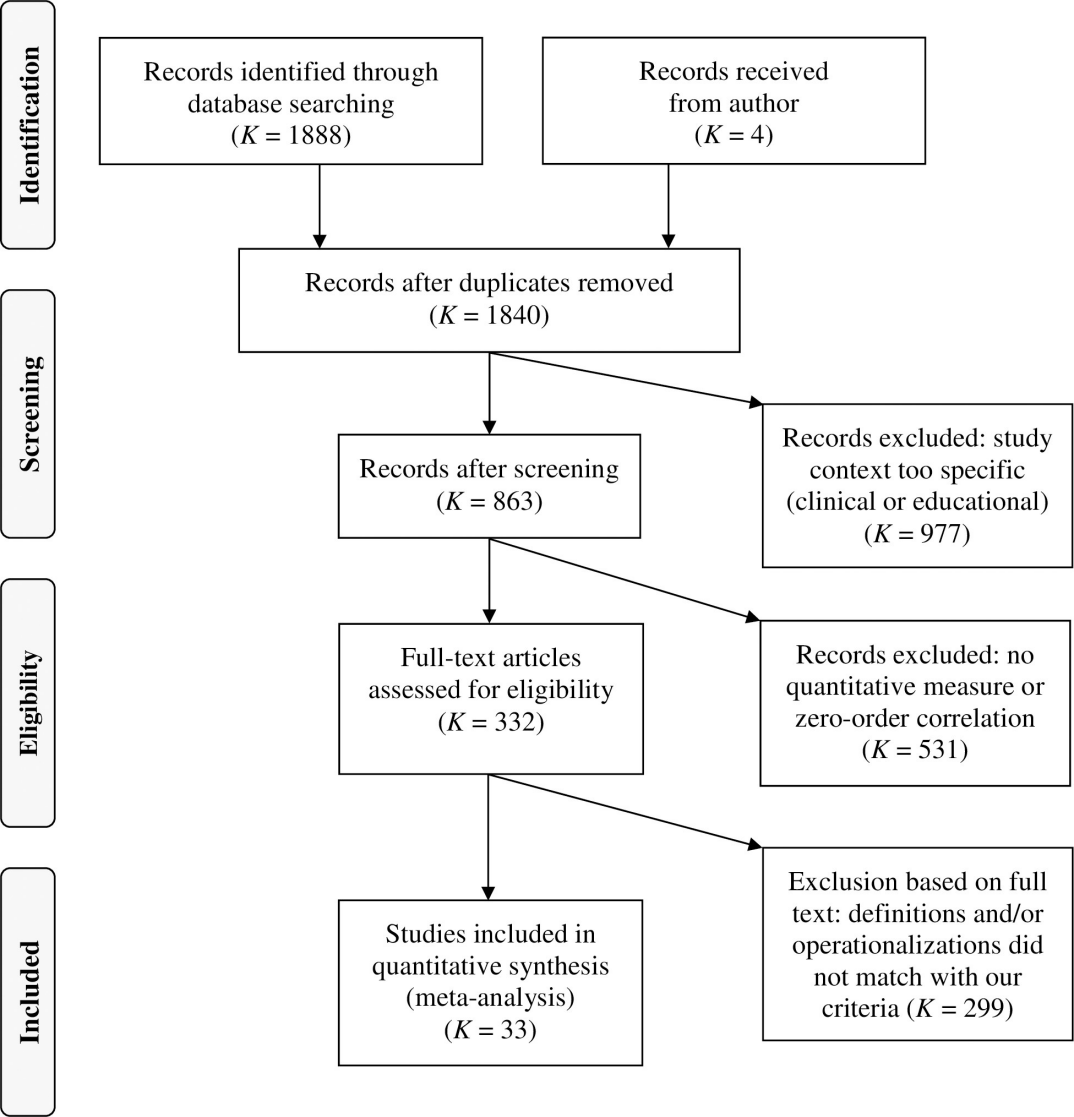
PEISMA flow diagram for inclusion and exclusion criteria for the present meta-analysis.

Within the scope of a preliminary screening, we excluded duplicates and studies conducted in clinical or educational settings. The latter were excluded because the cognitive abilities required for planning evolve over the human lifespan [48]. The remaining 863 studies were deemed to be eligible if they (a) included a quantitative measure of team planning, (b) included a quantitative measure of team performance, and (c) reported the zero-order correlation, or if the size and direction of the zero-order correlation could be computed from the information presented. Team planning did not have to be designated as such, but had to comply with the definition given above. Therefore, we included the study by Maynard and colleagues [49] and coded preparation activities as planning. Studies that looked at team plans and their consequences but not at the planning process itself (e.g., [50]) were not included (see Fig 2 for the PRISMA flow diagram showing inclusion and exclusion criteria for our meta-analysis). 33 studies with 96 effect sizes were eligible for inclusion in the meta-analysis.

### Coding procedure

Studies were coded for measures of team planning and team performance, the zero-order correlation between these two, and their respective reliabilities. When no reliability measure was reported we used the mean across all the reliabilities documented within that study as an estimate. For moderator analysis, we coded relevant task factors, team factors, context factors, and methodology factors (for details see Table 1). The coding scheme also included methodological quality (for details, see next section). Three trained reviewers (other than the authors) used a standardized coding format to extract data from studies and coded a third of the studies separately and in duplicate. The resulting average interrater reliability, as indicated by Cohen’s kappa coefficient, was κ = .86, generally regarded as “almost perfect” [51]. Any disagreements were resolved by consensus.

#### Methodological quality and statistical power assessment

We evaluated the methodological quality of the studies using 10 scored items, which were then combined to give a summary score (see S2 Appendix). On a scale from 1 (low) to 31 (high), the methodological quality of the studies ranged between 16.00 and 25.00, with a mean score of 21.04 (median = 21.00). This indicates that they were, on average, of medium methodological quality. We also assessed the post-hoc power of each study using G*Power [52].

### Statistical methods

We applied meta-analytic structural equation modeling (MASEM) using Mplus 8.4 [53]. MASEM combines the strengths of meta-analysis and structural equation modeling, including Bayesian estimation, allowing the modeling of error variance, evaluation of model fit, and examination of a complex set of theoretical hypotheses [54]. We conducted a one-stage three-level random effects model, which allowed each study to have its own population effect size [54]. This type of analysis therefore allowed us to account for nested effect sizes. We thereby estimated ‘regularized’ true study effects that are adjusted by within-study variability, showing better the true variation across studies and resulting in smaller and more uniform credible intervals compared to the sample mean [55]. In multilevel models it is assumed that the random effects are uncorrelated with the regressors. Violating this assumption creates endogeneity, which is a major threat to internal validity [56]. Following suggestions made by Antonakis and colleagues [56], we avoided this problem by adding interactions between the predictor and its cluster mean at level 1.

We used a Bayesian framework because of its advantages over conventional random-effects meta-analysis [57] and because it makes the parameter estimates and standard errors more reliable when fitting multilevel MASEM models with few higher level units (e.g., [58, 59]). We used standard settings for the prior distributions, which resulted in a flat, non-informative prior distribution. We employed four Markov chain Monte Carlo chains and set the number of iterations to 10,000. Of the 10,000 samples, every 20^th^ sample was stored and used for posterior inference to ensure model stability and avoid autocorrelation.

Model fit was indicated by the deviance information criterion (DIC) [60], which is a Bayesian generalization of the Akaike information criterion (AIC) and Bayesian information criterion (BIC) in ML, with low values indicating a better model fit. A potential scale reduction (PSR) criterion [61] (Gelman & Rubin, 1992) was checked, with a value close to 1 indicating that the between-chain variation is small relative to the within-chain variation and providing evidence of convergence. Finally, to evaluate the convergence behavior of the Markov chains, we checked trace plots to ensure there were no trends, spikes, or other irregularities. With all our models, we found there were only negligible irregularities or no irregularities at all. 90% credibility intervals that exclude zero indicate statistically significant one-sided relationships.

#### Estimation of overall effect size

Following Cheung [54], we estimated the average correlation for team planning and team performance at the within level in the three-level MASEM approach. This approach takes account of effect sizes nested within studies. We hereby corrected effect sizes for unreliability. We then transformed the effect sizes by dividing them by the known sampling variance of estimates, referred to as the “transformed variables approach” ([54] p. 314), which leads to a known variance of one. The levels were then specified as proposed by Cheung (2015) [54] as Level 2 (unique labels for effect sizes) and Level 3 (unique label for every study to provide information on how effect sizes are nested). The random slope was defined at the within level (Level 1). This leads to the following specification,

Level 1: $y_{ij}=\lambda_{ij}\cdot \sqrt{w_{ij}}+e_{ij}$,

Level 2: $\lambda_{ij}=f_{j}+u_{ij}^{\left(2\right)}$,

Level 3: $f_{j}=\beta_{0}+u_{j}^{\left(3\right)}$, where *y*_*ij*_ is the transformed effect size, $\sqrt{w_{ij}}$ the transformation variable, *e*_*ij*_ the transformed residual, *λ*_*ij*_ the true effect size for the *i*th effect size in the *j*th study, *f*_*j*_ the true effect size in the *j*th study, $u_{ij}^{\left(2\right)}\;\mathrm{and}\;u_{j}^{\left(3\right)}$ residual terms for respective levels, and β_0_ the average population effect ([54]; see also [62]). To characterize average effect sizes, we used the criteria outlined by Bosco and colleagues [63] for gauging small (below 0.09), medium (between 0.09 and 0.37), and large (greater than 0.37) effect sizes.

#### Identification of heterogeneity

Between-study heterogeneity was assessed in three ways. First, to determine whether there was substantial between-study variability in effect sizes that was not due to sampling error, we computed Cochran’s *Q*. Second, we computed $\tau_{\left(2\right)}^{2}\;\mathrm{and}\;\tau_{\left(3\right)}^{2}$, the heterogeneity variances at levels 2 and 3 respectively (i.e. $\mathrm{Var}(u_{ij}^{\left(2\right)})$ and $\mathrm{Var}(u_{j}^{\left(3\right)})$ respectively). Third, *I*^2^ was computed to examine whether the heterogeneity was substantial. Low, moderate, and high levels of heterogeneity in observed effect sizes that are not due to sampling error are indicated by *I*^2^ levels of 25%, 50%, and 75% respectively [64].

### Hypothesis testing

Consistent with the literature [65, 66] we used latent meta-regression within the three-level MASEM approach instead of subgroup analysis to clarify the relationship between multiple moderators and effect size. As moderators might be statistically confounded, multivariate techniques allow one to control for spurious influences [67]. The three-level random-effects meta-analysis was therefore extended to a mixed-effects model [54]. We then undertook a stepwise test of Research Questions 1a–d; to arrive at the most robust set of moderators, we used a series of latent multiple meta-regression analyses to identify significant predictors first in each category, then across categories using only those moderators which were initially significant. Specifically, the stepwise approach was applied to (a) reduce capitalization on chance and avoid an inflated Type I error rate, which can occur in separate linear regression analyses [68], (b) identify multicollinearity among subsets of correlates moderators, which can lead to inflated standard errors and biased estimates, and (c) identify a small number of moderators with the most explanatory power for our outcome. In step 1 we looked for multicollinearity among variables by examining bivariate zero-order correlations and we excluded any variables that correlated above the critical limit of .5 [69]. As a result, two variables (team familiarity and guided planning) were omitted from subsequent analyses. In addition, one moderator (knowledge distribution) was omitted due to the lack of variance reported in reviewed studies. In step 2 we performed four separate multiple regressions, with task factors (type of task, task interdependence, time constraints, and task complexity), team factors (authority, team size, skill level, and skill differentiation), context factors (planning session, task familiarity, and type of planning), and methodological factors (type of study, dimension of planning, study setting, type of planning data, and type of performance data) as the moderators. In step 3 we analyzed significant moderators of the step 2 analyses jointly in a multiple moderator model to examine the specific contribution of the most relevant moderators.

To test Hypothesis 2, we employed a latent class analysis (LCA) [70], applying a latent mixture modeling approach using the robust maximum likelihood estimator in Mplus 8.4. A latent mixture modeling approach offers many advantages over traditional cluster techniques, including that it enables rigorous statistical tests to be undertaken to assess model fit, provides formal criteria for making decisions about the adequate number of clusters, and allows different distributional forms to be used. We tested a series of unconditional LCA models, starting with a one-class model and then successive models with one additional class. We estimated the 10 best of 1,000 random starts with 40 iterations and checked that the results were sensitive to the number of random starts for the k class model. For class enumeration, the criteria we used were parsimony, substantive consideration, and fit indices. The information criteria were the AIC, consistent AIC (CAIC), BIC, and sample-size-adjusted BIC (SABIC) and likelihood ratio tests included the bootstrap likelihood ratio test (BLRT) and Lo–Mendell–Rubin likelihood ratio test (LMR-LRT). Classes that represented less than 5% of the sample were considered unacceptable (cf. [71]). We also checked the entropy values, which represent the quality of classification of individuals into latent classes. Entropy values over 0.8 indicate a good separation of the latent classes. The final model was evaluated in terms of its parsimony, theoretical justification, and interpretability [71, 72].

#### Statistical outliers

Based on our theoretical underpinnings which we addressed in the derivation of Hypothesis 2, we regard influential cases that appear to deviate from the overall patterns in data not primarily as statistical outliers but as substantial effects. Following the roadmap recommended by Gibbert and colleagues [73], we therefore did not remove influential cases but kept them in the analysis. However, as statistical outliers can cause serious problems in statistical analyses, we followed the rules for identifying influential cases in LCA modeling. Consistent with recommended best practice [74], we checked statistical outliers that had either a very small class to be extracted [75] or that did not make substantive sense in terms of parameter estimates [76]. There were no statistical outliers.

#### Statistical power

The post-hoc power of the three-level meta-analysis was assessed using Monte Carlo simulations, with estimates from our data as the population parameter values [59]. The robustness of the power estimates was checked using different seeds and 1,000 repetitions for each seed. The post-hoc power ranged between .986 and .990, which indicates excellent power to find moderate effect sizes (cf. [63]).

#### Sensitivity analysis

We explored the robustness of the observed outcomes by determining whether the main effect results were biased, due to the use of non-informational priors [77]. For this purpose, we performed supplementary analyses using vague priors for respective parameters—that is, normally distributed priors for location parameters with an expected value of zero and a variance of 10^10^ and inverse gamma priors for variances. The parameters of the inverse gamma distribution were set to 0.001, 0.01, and 0.1. In addition, findings from meta-analyses might also be biased due to the inclusion of low-quality and/or underpowered studies. Therefore, we assessed both the methodological quality and the post-hoc achieved power of each study (median = 0.42, range: 0.05 to 1.00). As proposed by Coyne and colleagues [78], we examined whether the findings were robust when studies with less than 50% power were excluded from the analysis. Finally, we used the methodological quality and statistical power of studies as moderating variables of the overall effect size to provide statistical evidence of possible bias.

## Results

**Fig 3:**
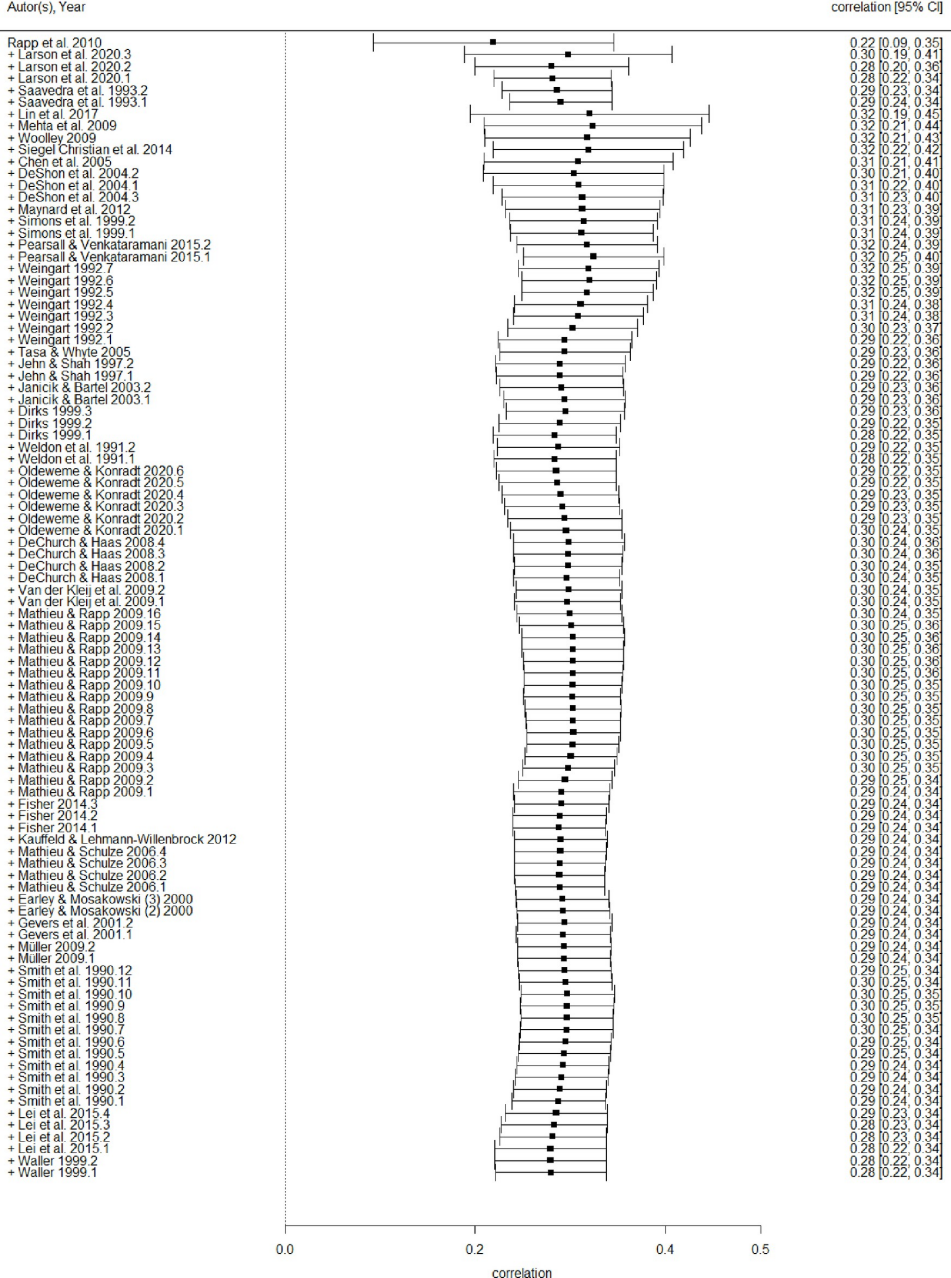
Cumulative forest plot arranged by study size, from highest to lowest.

Fig 3 displays the cumulative forest plot of the 80 effect sizes included in the meta-analysis, sorted from the largest to the smallest samples. Inspection of the overall effect sizes and their CIs indicated a very small drift of about 0.03, indicating no relationship between study size and effect size (cf. [79]). Hypothesis 1 predicted that team planning would be positively related to performance. Controlling for cluster means of Level 1 variables we found a positive significant relationship between team planning and performance (ρ = .31, *PSD* = 0.06, 95% CI [.20, .42], *p* < .001, *k* = 96 nested in *K* = 33 studies), supporting our hypothesis. The cluster mean of the Level 1 variables had no significant effect on the relationship (*B* = -0.28, *PSD* = 31.80, *ns*.), which indicated that there was no endogeneity bias in our model. Substantial heterogeneity was observed, as indicated by a significant Cochran’s *Q* (*Q* = 355.02, *p* < .001), a low to moderate *I^2^* (*I^2^* = 73.20%, *p* < .001), and significant heterogeneity variances at Level 2 and Level 3 ($\tau_{\left(2\right)}^{2}$ = .024, *p* = .003; $\;\tau_{\left(3\right)}^{2}$ = .024, *p* < .001). Hence, the findings suggest that variance across primary studies was not only due to sampling error, providing a rationale for further moderator and latent class analyses.

**Fig 4:**
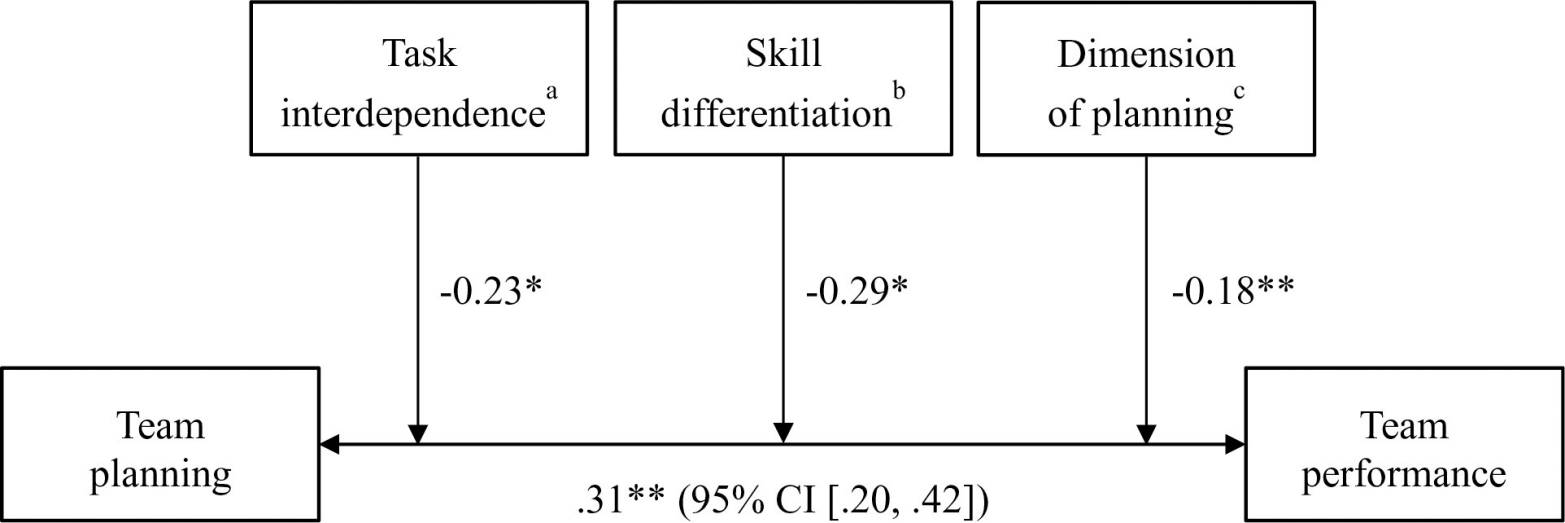
Summary of study results. Note ^a^ 1 = low, 2 = medium, 3 = high. ^b^ 1 = low, 2 = medium. ^c^ 1 = qualitative, 2 = quantitative. *p < .05. **p < .01.

Bivariate correlations of moderators are shown in Table 2. A stepwise meta-regression procedure was used to address Research Questions 1a–d to determine whether the association between team planning and performance varies according to task, team, context, and methodological factors (Table 3). Moderator analyses revealed that task interdependence (*B* = -0.23, *PSD* = 0.11, *p* = .02), skill differentiation (*B* = -0.29, *PSD* = 0.12, *p* = .01) as well as dimension of planning (*B* = -0.18, *PSD* = 0.06, *p* < .01) moderated the overall. This indicated that the effects were significantly larger when task interdependence was moderate compared to high (there were no studies with low/absent task interdependence), skill differentiation was low compared to moderate skill differentiation (there were no studies with high skill differentiation), and when the quality of team planning was assessed compared to the quantity of planning. A summary of study results regarding Hypothesis 1 and Research Question 1a-d can be found in Fig 4.

**Table 2:** Assessment of multicollinearity: Correlations between moderators.

		*M* (*SD*)	1	2	3	4	5	6	7	8	9	10	11	12	13	14	15	16	17
*Task factors*																			
1	Type of task	1.10 (0.40)																	
2	Task interdependence^b^	2.15 (0.35)	.12																
3	Time constraints	1.11 (0.32)	-.09	.22*															
4	Task complexity	2.28 (0.69)	-.22*	-.08	.28**														
*Team factors*																			
5	Authority	2.76 (0.43)	-.04	-.50**	.05	.21													
6	Team size	4.27 (3.32)	-.18	-.34**	-.17	-.01	.08												
7	Team familiarity ^a^	1.27 (0.64)	.08	.20	.68**	.14	-.08	-.20											
8	Skill level	1.82 (0.66)	-.09	-.16	.49**	.59**	-.00	.19	.61**										
9	Skill differentiation	1.11 (0.32)	-.09	.22*	.59**	.33**	-.11	-.21	.68**	.59**									
*Context factors*																			
10	Planning session	1.47 (0.50)	.28**	.20*	.19	.04	-.24*	.38**	.45**	.44**	.38**								
11	Task familiarity	1.77 (0.69)	-.14	-.12	.50**	.62**	.11	.26*	.60**	.90**	.55**	.47**							
12	Type of planning	1.63 (0.84)	.30**	.24*	.03	-.40**	-.28*	-.48**	.03	-.23*	.02	.15	-.26*						
13	Guided planning ^a^	1.52 (0.50)	.20	.22*	.35**	-.00	-.33**	.38**	.41**	.41**	.35**	.78**	.41**	.08					
*Methodological factors*																			
14	Type of study	1.90 (0.40)	.07	-.19	-.57**	-.05	.11	.10	-.85**	-.47**	-.57**	-.28**	-.47**	.15	-.25*				
15	Dimension of planning	1.40 (0.63)	.17	.26*	.06	-.46**	-.21	-.12	.11	-.05	.14	.12	-.07	.12	.25*	-.06			
16	Study setting	2.97 (1.18)	-.02	.11	-.02	.45**	.06	.45**	-.33**	.21*	-.02	-.08	.18	-.36**	-.03	.18	-.14		
17	Type of planning data	1.71 (0.81)	-.10	-.03	-.11	.32**	.21	-.45**	-.13	.08	.01	-.49**	.05	-.25*	-.50**	.07	-.02	.39**	
18	Type of performance data	1.25 (0.50)	-.08	.09	.24*	.16	-.19	-.28*	.45**	.43**	.41**	.42**	.40**	.40**	.32**	-.37**	.01	-.31**	-.23*

*Note*. *k* = 38 to 80. Italicized estimates are not statistically different from zero at α = .05 (two-sided). See Table 1 for coding of moderators.

^a^ These variables were omitted from subsequent analyses due to multicollinearity.

^b^ Task interdependence was binary, as no study was coded with a 1 = “low”. Knowledge distribution was omitted due to insufficient variance.

*p < .05.

**p < .01.

**Table 3:** Results of the Bayesian stepwise meta-regression.

Predictors	Model 1		Model 2		Model 3		Model 4			Model 5	
	B (*PSD*)	*p*	B *(PSD)*	*p*	B *(PSD)*	*p*	B *(PSD)*	* *	*p*	B *(PSD)*	*p*
*Task factors* (*k* = 96; DIC = 329.992)											
Type of task	-0.07 (0.08)	.21									
Task interdependence	**-0.25** (0.11)	.01									
Time constraints	-0.12 (0.12)	.15									
Task complexity	-0.05 (0.07)	.23									
*Team factors* (*k* = 50; DIC = 171.241)											
Authority			-0.07 (0.11)	.27							
Team size			**0.09** (0.05)	.04							
Skill level			-0.01 (0.07)	.44							
Skill differentiation			**-0.24** (0.15)	.05							
*Context factors* (*k* = 81; DIC = 278.258)											
Planning session					-0.01 (0.13)	.47					
Task familiarity					-0.05 (0.08)	.28					
Type of planning					0.04 (0.07)	.27					
*Methodological factors* (*k* = 93; DIC = 316.551)											
Type of study							0.12 (0.11)	.12			
Dimension of planning							**-0.16** (0.06)	.00			
Study setting							-0.05 (0.04)	.13			
Type of planning data							-0.05 (0.08)	.24			
Type of performance data							0.07 (0.08)	.17			
*Overall* (*k* = 62; DIC = 209.064)											
Task interdependence										**-0.23** (0.11)	.02
Team size										-0.01 (0.02)	.23
Skill differentiation										**-0.29** (0.12)	.01
Dimension of planning										**-0.18** (0.06)	.00

*Note*. *k* = number of study estimates used in analysis. Significant parameter estimates in bold face. *PSD* = Posterior *SD*. See Table 1 for coding of moderators.

Hypothesis 2 predicted there would be two classes with distinct effect sizes in terms of the relationship between planning and performance in teams. As shown in Table 4, the two-class model outperformed the one-class model, as indicated by the information criteria and BLRT. SABIC, the most effective model selection index for class enumeration in growth mixture models and best the index to use when the sample size is small [80], also indicated a better model fit. However, the two-class model produced a small class (*k* = 4), which violates the assumption of there being a substantial class size [71]. Thus, Hypothesis 2 was rejected. This result indicates that there is no substantial variation in the relationships between planning and performance. Rather, we can assume a homogeneous class of relationships in the data, which supports our previous finding of a moderately sized positive association between team planning and performance.

**Table 4:** Fit of growth mixture models.

No. of classes	Likelihood ratio tests, *p* values		Information criteria				Entropy
	BLRT	LMR-LRT	CAIC	AIC	BIC	SABIC	
1	−	−	396.11	388.98	394.11	387.79	−
2 ^a^	< .001	< .001	390.13	375.88	386.13	373.51	0.94

*Note*. BLRT = bootstrap likelihood ratio test; LMR-LRT = Lo–Mendell–Rubin likelihood ratio test; AIC = Akaike information criterion; CAIC = consistent AIC; BIC = Bayesian information criterion; SABIC = sample-size-adjusted BIC.

^a^ Class 2 with *k* = 4.

### Sensitivity analyses

We performed sensitivity analyses to explore the robustness of the meta-analytic estimates. First, the previous sensitivity analyses indicated no bias resulting from the use of non-informational priors. Differences between the estimates were found only in the third decimal, suggesting that the results were stable when using non-informational rather than vague informational priors. To check whether the relationship found was robust when we accounted for methodological quality and achieved power of primary studies, we undertook two meta regressions. The findings suggested that the relationship was not biased by the quality of primary studies (*B* = -0.02, *PSD* = 0.01, *ns*.) but was biased significantly by achieved power (*B* = 0.58, *PSD* = 0.06, *p* < .001), as elevated relationships were found in studies with higher power. To clarify this, we tested whether the findings were robust when only primary studies with a power higher than 0.5 (*k* = 42) were used in the analysis. This led to a larger mean effect size point estimate and CI (ρ = .40, *PSD* = 0.07, 95% CI [.27, .54], *p* < .001) in studies with higher achieved power. In contrast, we found a smaller, significant effect size in studies with a power less than or equal to 0.5 (*k* = 54, *PSD* = 0.07, 95% CI [.01, .30], *p* = .02). Thus, the magnitude of the effect size was sensitive to power limitations, and the smaller effect size estimate for the full set of studies constituted the lower point estimator of the true effect.

Furthermore, as Table 3 indicates, Study Setting and Type of Study do not influence or bias the results of the meta-analysis in a multivariate setting. To check if this holds true for the univariate cases of Study Setting and Type of Study as well as Sample we conducted univariate subgroup analyses revealing no significant moderation for all three variables.

## Discussion

The aim of this study was to meta-analyze studies on the effectiveness of team planning for team performance and to identify moderator variables that affect the effect sizes obtained. In addition, while most of the literature to date has focused on the positive effects of planning on performance, we also examined samples with null association between planning and performance.

Our findings provide clear evidence of a moderately sized positive association between team planning and performance, which corroborates existing theoretical and empirical literature [e.g., 5–7]. There are three ways in which these findings are substantiated. First, by using a mixed linear model to estimate true study effects, we accounted for within-study variability. These regularized model estimates [55] provide a better indication of the true variation across studies and result in smaller and more uniform credible intervals compared to the sample mean. Second, the sensitivity analyses and publication bias testing revealed that our findings were generally robust, except in the case of power. Because the effect size estimate across studies with adequate power led to elevated effects, our general finding represents the lower boundary of the estimate. Third, the methodological quality of studies did not substantially alter the result. However, as the parameter estimates were not inflated by low-power studies, the effect size estimate for the full set of studies represents a rather conservative point estimate of the true effect.

The results of this study also suggest that the effect size is only marginally affected by the boundary conditions. Apart from task interdependence, skill differentiation, and dimension of planning (quality vs. quantity), none of the other moderators discussed in substantive theory [8, 26, 27] affected the mean effect sizes. This justifies the conclusion that our result is generalizable across different tasks, team settings, and contexts. Our finding—that the dimension of planning moderated the effect sizes and was associated with larger effect sizes when quality rather than quantity of planning was assessed—is in line with the notion that the sheer amount of planning does not necessarily lead to higher performance levels if the quality of planning is poor or the plan itself is unsuitable [40] A few studies of transition processes also demonstrated the importance of the quality, as opposed to the quantity, of team processes (e.g., [81, 82]). For example, Otte and colleagues [81] revealed that the amount of team reflection was negatively related to performance improvement and the quality of team reflection was positively related to it when being considered simultaneously. Likewise, Santos and colleagues [82] found that when a team’s mental model lacks quality, a highly similar mental model may be disruptive to team functioning. Furthermore, we found that task interdependence negatively moderated effect sizes, meaning that moderate levels of task interdependence were associated with a greater relationship between team planning and performance than high levels of task interdependence. However, there were no studies coded with a 1 = “low”, meaning that the moderator was dichotomous. Hence, we cannot draw any conclusions about the relationship between team planning and performance when task interdependence is low or even absent. The findings together with past research [83] might suggest a quadratic association, meaning that at least a moderate task interdependence is needed for planning to be effective at the first glance, but very high levels of task interdependence might be detrimental to the functioning of team planning. Hence, findings should be taken with caution and further research on team planning in situations where task interdependence is low as well as the investigation of possible quadratic or curvilinear associations is needed.

In addition, our results showed that when the level of team members’ specialized knowledge or functional capacity was low, effect sizes were higher [34]. These results can be explained by the fact that members in teams characterized by low skill differentiation do not have specific roles associated with their abilities. Consequently, those teams must determine their specific and distinctive roles, determine objectives and tasks. However, there were no studies with high levels of skill differentiation, hence we advocate future research to investigate the relationship in those situations.

There are several possible reasons for the marginal influence of moderators. First, the moderating effects found in previous studies are based on univariate analyses, which, when the moderators overlap empirically, can result in parameter bias [67]. Because we controlled for spurious influences, our results are more conclusive with respect to potential statistical confounding. Also, several primary studies were underpowered, which could have led to an increased risk of Type I error and inflated effect-size estimates [84]. This risk is particularly present in team studies, where the sample size shrinks because team member responses are aggregated to the team level. As we aggregate across studies, our results at the meta-analytic level provide the largest statistical power of true moderator effects being the first one to reach an adequate level [85]. One must also keep in mind that our comparisons are non-experimental, which bears the risk that our results may be confounded by other variables. Finally, the heterogeneity of many moderator variables was restricted, often limited to very few categories, and only a few (or no) studies were available, which might be a threat to validity [79]. Although a common practice in meta-analysis, one must be cautious of generalizing findings unless there is sufficient causal evidence to support the results.

We also demonstrated that all effect sizes have true positive effects. This finding contradicts the results of previous research (e.g., [12, 13]) and confirms our general result. Nevertheless, taking into account heterogeneity in the variance of unobserved effects could open up a new avenue for research.

Finally, the current review also enables us to identify critical gaps in the literature. Very few of the studies (e.g., [86, 87]). included in this review examined temporal aspects of change in team planning. Adopting a temporal lens in research and looking at the duration, frequency, timing, and sequence of planning processes [88, 89] would improve the literature substantially. We thus advocate studies with strong designs, such as intensive longitudinal designs [90] involving 20 or more waves of data collection, as this may help to overcome this limitation and provide a better understanding of the team-specific dynamics (cf. [91]). It is also noteworthy that only a few studies differentiated between the quality and quantity of team planning. As mentioned above we found a moderating effect of the dimension of planning (assessment of quality or quantity), which might indicate a similar functioning as the aforementioned transition processes. However, it is not fully understood how these dimensions are related. In this meta-analysis we were not able to analyze the quantity and quality of team planning simultaneously, like, for example, Otte and colleagues [81] did with team reflection. Future research should thus expand on the current findings by assessing how the quality and quantity of planning in combination affect team outcomes (see also [92]).

There are several research and managerial implications from the results of this study. The positive effect of team planning on performance was found to be robust with respect to nearly all moderator variables, indicating general importance of planning to improve performance. This applies in particular when team members do not have specific and distinctive roles. This suggests that managers should encourage their teams to plan and create a supportive environment by establishing guidelines for effective planning, promote appropriate communication methods, and make use of project management tools. Furthermore, as the findings for the dimension of planning suggest, the quality of planning rather than the sheer amount of planning should be emphasized within planning activities. The robust effect size estimator provides valid information to calculate the economic benefits and cost of interventions in teams that are seeking to improve their planning [93]. Finally, our findings provide useful information for researchers to select appropriate sample sizes for future studies and determine realistic prior distribution in Bayesian models, which outperform noninformative, weakly informed, and diffuse priors in model parameter estimates [57].

### Limitations and future directions

This study and its results have some limitations, which suggest potential directions for future research. The main limitations of this meta-analysis pertain to the methodological limitations of primary studies. First, only a relatively small number of empirical studies were available, and the methodological quality in terms of the lack of randomized controlled trials was not convincing, which limits the validity and generalizability of our results. Another limitation is that the findings might be biased by the power achieved in primary studies [78]. Less than half of the effect sizes (*k* = 42) analyzed were from studies that had achieved adequate post-hoc power. We found that studies that were adequately powered showed both a larger positive effect and a narrower confidence interval compared to the main effect. For this reason, we advocate future research to increase study power not only by referring to the omnipresent call for larger sample sizes. However, as applied researchers are compelled to limit their sample sizes for economic and other reasons, alternative ways of increasing power are to use within-subject or longitudinal research designs, extreme groups, planned missing designs, or potential covariates in experimental studies (see [94], for a review). Another way to increase power is to decrease standard errors by adding more explanatory variables (i.e., covariates) that reduce error variance in the criteria.

## Conclusion

With our study, we aimed to examine the extent of the association between team planning and team performance and to expand the rather narrow focus of much research on the positive effects of planning, enabling us to provide a more nuanced view. Based on the data available, we found an overall positive medium effect size, which was only marginally affected by a wide range of contexts and settings.

## Supporting information

S1 Checklist PRISMA 2020 checklist. (DOCX)

S1 Appendix Descriptive statistics of studies used in the meta-analysis [2, 4, 5, 12, 13, 18, 24, 36, 41, 49, 83, 87, 95–113]. (DOCX)

S2 Appendix Checklist for the assessment of the methodological quality of the studies reviewed. (DOCX)
