# Supplementary material for: Planning and performance in teams: A Bayesian meta-analytic structural equation modeling approach
Source: PLoS One. 2023 Jan 13;18(1):e0279933. doi: 10.1371/journal.pone.0279933 (PMC9838875; doi:10.1371/journal.pone.0279933)
Supplement: S1 Appendix — (DOCX) [file pone.0279933.s002.docx]

**Appendix A**

*Descriptive Statistics of Studies used in the Meta-Analysis*

| Study | *N* Teams | Average team size | Range of  Team size | Average age | % males | Sample | Type of study | Study setting |
| --- | --- | --- | --- | --- | --- | --- | --- | --- |
| Chen et al. (2005) [36] | 78 | 2 | 2 | 20 | 74.00 | Students | Experimental | Hypothetical |
| DeChurch & Haas (2008) [2] | 38 | NA | 3–4 | NA | NA | Students | Experimental | Hypothetical |
| DeShon et al. (2004) [95] | 75 | 3 | 3 | NA | 44.00 | Students | Experimental | Hypothetical |
| Dirks (1999) [96] | 42 | 3 | 3 | NA | NA | Students | Experimental | Hypothetical |
| Earley & Mosakowski (2000) (a) [97] | 23 | 4 | 4 | 43 | 76.00 | White collar | Experimental | Hypothetical |
| Earley & Mosakowski (2000) (b) [97] | 24 | NA | 6–8 | 28.54 | 68.00 | Students | Correlational | Real-life |
| Fisher (2014) [5] | 32 | NA | 2–8 | 24.15 | 52.20 | Students | Experimental | Hypothetical |
| Gevers et al. (2001) [98] | 22 | NA | 3–6 | NA | NA | Students | Correlational | Real-life |
| Janicik & Bartel (2003) [4] | 48 | NA | 4–5 | NA | NA | Students | Experimental | NA |
| Jehn & Shah (1997) [99] | 53 | 3 | 3 | 28.21 | 62.00 | White collar | Experimental | Hypothetical |
| Kauffeld & Lehmann-Willenbrock (2012) [100] | 30 | NA | NA | NA | 90.60 | Blue collar | Correlational | Real-life |
| Larson et al. (2020) [101] | 214 | 4.85 | 3-6 | NA | 52.00 | Students | Correlational | Real-life |
| Lei et al. (2016) [12] | 11 | 2 | 2 | 40 | 100.00 | White collar | Experimental | Hypothetical |
| Lin et al. (2017) [102] | 92 | NA | 5–NA | NA | 47.77 | White collar | Correlational | Real-life |
| Mathieu & Rapp (2009) [103] | 32 | NA | NA | 28.50 | 57.00 | Students | Experimental | Hypothetical |
| Mathieu & Schulze (2006) [104] | 29 | NA | 3–4 | 22 | 59.00 | Students | Experimental | Hypothetical |
| Maynard et al. (2012) [49] | 60 | 5.80 | 3–52 | 43 | 54.00 | White collar | Correlational | Real-life |
| Mehta et al. (2009) [24] | 91 | 5 | NA | 22.20 | 62.00 | Students | Experimental | Hypothetical |
| Müller (2009) [105] | 20 | 3 | 3 | 23.62 | 91.70 | Students | Experimental | Hypothetical |
| Oldeweme & Konradt (2020) [106] | 40 | 3 | 3 | 21.16 | 19.00 | Students | Experimental | Hypothetical |
| Pearsall & Venkataramani (2015) [107] | 56 | 5 | 5 | 21.50 | 53.00 | Students | Experimental | Hypothetical |
| Rapp et al. (2010) [108] | 218 | 5 | NA | NA | 48.00 | White collar | Correlational | Real-life |
| Saavedra et al. (1993) [83] | 118 | 3 | 3 | NA | NA | Students | Experimental | Hypothetical |
| Siegel Christian et al. (2014) [109] | 78 | 4 | 4 | 21 | 40.60 | Students | Experimental | Hypothetical |
| Simons et al. (1999) [110] | 57 | 6.06 | NA | NA | NA | White collar | Correlational | Real-life |
| Smith et al. (1990) [41] | 16 | 18.50 | NA | NA | NA | Students | Experimental | Hypothetical |
| Tasa & Whyte (2005) [111] | 54 | 3 | 3 | 22.10 | 48.15 | Students | Experimental | Hypothetical |
| van der Kleij et al. (2009) [112] | 36 | 2 | 2 | 22.15 | 50.00 | Students | Experimental | Hypothetical |
| Waller (1999) [13] | 10 | 3 | 3 | NA | 100.00 | White collar | Experimental | Hypothetical |
| Weingart (1992) [18] | 56 | 4 | 4 | NA | NA | Students | Experimental | Hypothetical |
| Weldon et al. (1991) [113] | 40 | 3 | 3 | NA | NA | Students | Experimental | Hypothetical |
| Woolley (2009) [87] | 90 | 3 | 3 | NA | NA | Students | Experimental | Hypothetical |

*Note*. NA = information not available.
