# Supplementary material for: Planning and performance in teams: A Bayesian meta-analytic structural equation modeling approach
Source: PLoS One. 2023 Jan 13;18(1):e0279933. doi: 10.1371/journal.pone.0279933 (PMC9838875; doi:10.1371/journal.pone.0279933)
Supplement: S2 Appendix — (DOCX) [file pone.0279933.s003.docx]

**Appendix B**

*Checklist for the Assessment of the Methodological Quality of the Studies Reviewed*

| *Sampling and representativeness* | |
| --- | --- |
| 1 | Was the response rate reported? (0–40, 41–60, 61–80, 81–100) ^a^ |
| 2 | Are the individuals selected to participate in the study likely to be representative of the target population? (sex, age, type of personnel, operational context) ^a^ |
| 3 | Were the study subjects and setting described in detail? ^a^ |
| 4 | Are dropout/missing values described, analyzed, and discussed? ^b^ |
| *Statistical analyses* | |
| 5 | Is the statistical power calculated? ^b^ |
| 6 | Are the statistical methods appropriate for the study design? ^a^ |
| *Measurement (reliable and valid)* | |
| 7 | How was team planning measured? ^a^ |
| 8 | How was team performance measured? ^c^ |
| *Design and fidelity* | |
| 9 | Design used (implementation/type of design: cross-sectional, longitudinal) ^d^ |
| 10 | Relevant discussion of study limitations ^a^ |

*Note.* ^a^ 0 = not reported/unsatisfactory, 1 = poor, 2 = satisfactory, 3 = good, 4 = excellent. ^b^ 0 = not reported, 1 = reported. ^c^ 1 = self-report, 2 = time measure, 3 = external assessment, 4 = objective data. ^d^ 0 = cross-sectional, 1 = longitudinal.
